# Rhabdomyolysis as Potential Late Complication Associated with COVID-19

**DOI:** 10.3201/eid2610.202225

**Published:** 2020-10

**Authors:** Kok Hoe Chan, Jihad Slim

**Affiliations:** Saint Michael’s Medical Center, New York Medical College, Newark, New Jersey, USA

**Keywords:** rhabdomyolysis, SARS-CoV-2, COVID-19, respiratory infections, severe acute respiratory syndrome coronavirus 2, 2019 novel coronavirus disease, coronavirus disease, zoonoses, viruses, coronavirus

**To the Editor:** Jin and Tong described a patient with severe coronavirus disease (COVID-19) in whom rhabdomyolysis developed on day 9 of hospitalization (*1*). The interplay between severe acute respiratory syndrome coronavirus 2 and rhabdomyolysis is not yet understood; we consider possible etiologies for this case of rhabdomyolysis.

We reported 2 case-patients with COVID-19 who also had weakness and elevated creatinine kinase levels (but no respiratory symptoms) (*2*). As part of his COVID-19 treatment regimen, the patient reported by Jin and Tong received lopinavir and meropenem, which can cause rhabdomyolysis (*3*,*4*). Meropenem is associated with rhabdomyolysis by inducing severe hypomagnesemia and hypokalemia; it would be helpful to know the trends in the patient’s electrolytes before rhabdomyolysis developed (*3*). A cytokine storm might also have caused this complication because rhabdomyolysis developed on day 15 of COVID-19 symptoms and coincided with the peak of inflammatory markers (C-reactive protein). On the other hand, the combination of hypoxia and hypercoagulability might have induced an ischemic event that inhibited blood flow to the involved muscles, triggering rhabdomyolysis.

Clinicians treating rhabdomyolysis concurrent with COVID-19 must assess the many differential diagnoses, including severe acute respiratory syndrome coronavirus 2–induced myositis, reactions to medication, cytokine storm, hypoxia, or a thromboembolic event. This differential diagnosis is crucial because each condition has a distinct therapeutic approach.
